# Case Report: Prenatal genetic analysis of a rare fetus with 45, X/46, X, dic r (Y; Y)/46, X, r(Y) karyotype

**DOI:** 10.3389/fgene.2026.1758508

**Published:** 2026-03-30

**Authors:** Guosheng Deng, Xiafei Liang, Yuqing Lai, Jujie Song, Jinjie Pan, Yinghong Lu, Lili Li, Yunning Liang

**Affiliations:** 1 Department of Laboratory Medicine, Yulin Maternal and Child Health Hospital, Yulin, Guangxi, China; 2 Obstetrics and Gynecology Outpatient Department of Yulin Maternal and Child Health Hospital, Yulin, Guangxi, China; 3 Department of Surgery, Yulin Maternal and Child Health Hospital, Yulin, Guangxi, China

**Keywords:** chimerism, chromosome karyotype analysis, CNV-seq, fetus, fluorescence *in situ* hybridization

## Abstract

**Objective:**

To perform a genetic analysis of a rare complex chimeric fetus with a 45,X/46,X,dic r(Y; Y)/46,X,r(Y) karyotype, indicated by NIPT as having sex chromosome abnormalities but with normal ultrasound findings. This study underscores the critical role of integrating multiple molecular cytogenetic techniques in deciphering such complex cases, which is essential for accurate prognosis and personalized genetic counseling. The findings aim to deepen the understanding of genotype-phenotype correlations in rare chromosomal mosaicism and to guide clinical management.

**Method:**

Amniotic fluid was collected from a pregnant woman with an abnormal sex chromosome indicated by NIPT. Combined detection using G-banding karyotype analysis, fluorescence *in situ* hybridization (FISH), and low-depth whole-genome copy number variation sequencing (CNV-seq) techniques was performed. Simultaneously collect peripheral blood samples from the fetus’s parents for CNV-seq detection and paternal chromosomal karyotype analysis. The infant underwent comprehensive postnatal follow-up, including physical examination, growth assessment, developmental screening, sex hormone profiling, Y chromosome microdeletion testing, and scrotal ultrasound at 19 months of age.

**Result:**

The male fetus was confirmed to have a complex karyotype through combined analysis of chromosomal G-band technology, FISH, and CNV-seq. The findings included a dicentric ring Y chromosome with mosaicism for Yp and Yq deletions, as well as a 1.40 Mb duplication in the 7q11.23 region, resulting in the karyotype: 45,X[82]/46,X,dic r(Y; Y)(p11.31q11.23; p11.31q11.23)[13]/46,X,r(Y)(p11.31q11.23) [5]dn. The father’s karyotype was normal, suggesting a *de novo* mutation. Maternal CNV-seq was normal, while paternal CNV-seq identified the same 1.40 Mb 7q11.23 duplication, indicating paternal inheritance of this pathogenic variant. After genetic counseling, the parents proceeded with the pregnancy. On 27 June 2024, at 35^+5^ weeks of gestation, they gave birth to a live male infant naturally, with a length of 48 cm and a weight of 2800 g. No obvious abnormalities were observed in the appearance.

**Conclusion:**

The integration of G-banding, FISH, and CNV-seq enables accurate diagnosis of complex ring Y chromosome mosaicism, providing crucial information for genetic counseling and clinical management. The clinical phenotype depends on the ring chromosome’s structure, breakpoints, and the degree of mosaicism.

## Introduction

Ring chromosomes (RCs) refers to a chromosome with two distal segments that break once at each end, and the two broken ends of the centromere segment are reconnected in a circular shape, also known as the centromere ring; If two broken ends of a segment without centromeres are reconnected in a circular shape, it is called an acentric ring. The most commonly recorded ring chromosomes in humans today are r (Colindres et al.), r (Ravel and Siffroi, 2009), r (DuPont et al., 2024), r (Chong and Muir, 2025), r (Khudr and Benirschke, 1973), r (X), *etc.* R(Y) and dic r(Y; Y) are rare, and 45, X/46, X, dic r(Y; Y)/46, X, r(Y) chimeras are extremely rare in clinical and even prenatal diagnosis, and have not been reported in China. It is one of the disorders of sex development (DSD) caused by sex chromosome abnormalities, and its gonadal manifestations include mixed gonadal dysplasia and ovotesticular DSD (Pristyazhnyuk and Menzorov, 2018). Its incidence is 1/5,000 to 1/4,500 of newborns (Houk and Lee, 2008), which may be due to the simultaneous breakage of the long and short arms of the Y chromosome to form RCs. During cell mitosis, RCs can be involved in sister chromatid exchange, leading to the formation of double ring chromosomes, dicentric rings, or other complex structures. In addition, RCs are prone to loss during cell division and exhibit instability. Therefore, individuals with RCs often exhibit mosaicism, with some cells containing ring chromosomes, some containing double ring chromosomes, and some losing the ring chromosome entirely. The most common type of mosaicism is 45, X (Melzer et al., 1993). These patients, similar to those with 45, X/46, XY mosaicism, have a complex etiology and diverse clinical manifestations, which can range from Turner syndrome stigmata in females, ambiguous external genitalia, and infertility, to a completely normal male phenotype (Colindres et al.; Blanco et al., 2003). This study applied G-banding chromosome karyotyping analysis, FISH, and low depth whole genome CNV-seq techniques to deeply analyze the genetic characteristics and possible pathogenesis of this rare chromosomal disease, providing a basis for further understanding of this type of rare chromosomal disease.

## Case presentation

The pregnant woman is 32 years old and has no history of adverse pregnancy or childbirth. This test tube pregnancy is a singleton pregnancy in the uterus, with no special circumstances during early pregnancy, NT1.9mm, NIPT indicates sex chromosome abnormalities, 4D color ultrasound image at 18 weeks of pregnancy: BPD37mm, Head circumference 136mm, abdominal circumference 116mm, femur length 20mm, humerus length 20mm. On 23 February 2024, at 19 weeks of pregnancy, amniocentesis was performed in our hospital for prenatal diagnosis, chromosome karyotyping analysis, FISH, and CNV seq testing. The result of chromosome karyotyping analysis is 45, X[82]/46,X,dic r(Y; Y)(p11.31q11.23; p11.31q11.23)[13]/46,X,r(Y)(p11.31q11.23)[5]dn; The FISH results showed that the signal of the X chromosome centromere probe was normal, while the Y chromosome centromere probe exhibited signal abnormalities in some nuclei, manifested as signal loss, 1 signal, and 2 signals, which were consistent with the Y chromosome abnormalities found in G-banding karyotype analysis, further confirming the existence of a double centromere circular Y chromosome; CNV-seq detection showed a 1.40 Mb repeat region at q11.23 on chromosome 7; The chimeric deletion of 7.44 Mb region (copy number 0.7) at p11.31-p11.2, 11.40 Mb region (copy number 0.4) at q11.1-q11.223, and 0.28 Mb region (copy number 0) at q11.23 on the Y chromosome cover the entire region of the Y chromosome. The father of the fetus has a normal phenotype and normal chromosomal karyotype results, suggesting that the fetus’s karyotype is a *de novo* variation. The mother’s CNV-seq results are normal, while the father’s CNV-seq results show a 1.40 Mb duplication at chromosome 7q11.23, indicating that the fetus’s 7q11.23 microduplication syndrome is inherited from the father. Family history: no special remarks. After genetic counseling and fully informing the patient and her family about the condition and related risks, the couple ultimately chose to continue the pregnancy.

**FIGURE 1:**
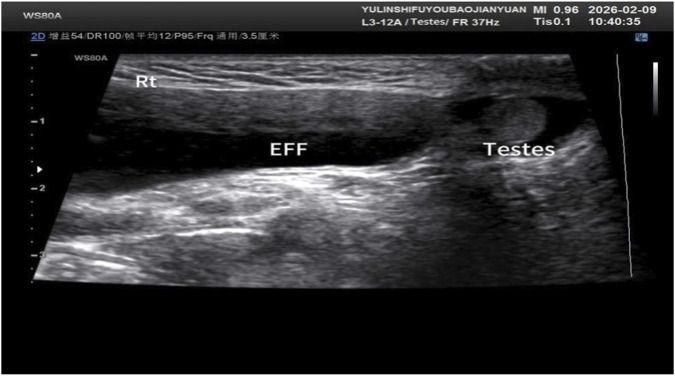
Color Doppler ultrasound examination of the scrotum and inguinal regions revealed bilateral cryptorchidism and a right funicular hydrocele.

The pregnancy resulted in the spontaneous vaginal delivery of a live male infant on 27 June 2024, at 35 weeks and 5 days of gestation. At birth, the infant measured 48 cm in length and weighed 2,800 g, with no obvious phenotypic abnormalities observed. Postnatal follow-up indicated that at birth, the infant’s length and weight were in the low-to-average range. While both parameters showed catch-up growth by 1 month of age, by 3 months of age, the infant’s weight had declined, approaching −3 standard deviations (SD) below the mean. From 3 to 8 months of age, linear growth demonstrated good catch-up, reaching an average level, and weight approached the average range. However, after 8 months of age, weight gain was unsatisfactory. By 1 year of age, weight had decreased to −2 SD (below average) and length had fallen to the low-to-average range, leading to a diagnosis of malnutrition (underweight). At a recall assessment at 1 year and 7 months of age, the child’s physical examination revealed a length of 76 cm (low-to-average, −2.79 SDS) and a weight of 9.4 kg (average to low, −1.91 SDS). Clinical manifestations included mild language developmental delay; although the child could walk independently, spontaneous speech was limited to a few reduplicated words and single syllables. Physical examination findings included a rounded nasal tip, a short philtrum, a unilateral single transverse palmar crease on the left hand, and a right inguinal mass, soft in consistency, measuring approximately 5 × 2 cm. Both testes were non-palpable in the scrotum. No intellectual disability was evident. Laboratory investigations, including sex hormone profiling, yielded results within normal ranges (Prog 0.1 ng/mL, E2 10 pg/mL, PRL 4.17 ng/mL, Testo 0.13 ng/mL, hFSH 0.87 mIU/mL, hLH 0.04 mIU/mL). Y-chromosome microdeletion analysis showed no deletions at the tested AZF loci: AZFa (sY86, sY84), AZFb (sY134, sY127), and AZFc (sY254, sY255). A color Doppler ultrasound of the scrotum and inguinal regions revealed a right funicular hydrocele and bilateral cryptorchidism, with the right testis measuring 12 × 10 × 6 mm and the left testis measuring 11 × 10 × 7 mm (Figure 1). Given the patient’s young age, long-term follow-up through puberty is warranted to assess gonadal development and future reproductive function.

## Methods

### Sample collection

After the pregnant woman and her family signed the informed consent form, the clinical physician will extracted 20–30 mL of amniotic fluid through abdominal puncture under ultrasound guidance. Amniotic fluid samples were divided into three parts: one for chromosome G-banding analysis after cell culture, one for FISH detection, and the other for DNA extraction for CNV-seq detection. Simultaneously, 2 mL of peripheral blood was collected from the pregnant woman into EDTA tubes for STR validation. Peripheral blood samples were collected from her husband for chromosome karyotyping analysis.

### Chromosome karyotype analysis

Amniotic fluid cells were conventionally cultured and harvested using a passaging method. Routine chromosome G-banding preparation was performed. Slides were automatically scanned using a Leica automated scanning microscope (USA) and its image analysis system. Karyotype analysis was performed using AutoVision® chromosome intelligent AI analysis software (Hangzhou Deshi Biotechnology Co., Ltd.), with 20–30 metaphase cells counted and 5 karyotypes analyzed. For suspected mosaic cases, the cell count was doubled or increased to 100 or more. The karyotype description was based on the International System of Nomenclature for Human Cytogenomics (ISCN 2020).

### FISH testing

Based on the principle of complementary pairing of DNA bases, FISH was performed using the directly labeled CSPX (green)/SRY (red) probe set, which targets the Xp11.1-q11.1 and Yp11 regions, respectively. Abnormalities of the target chromosomes were determined based on the number and position of the differently colored fluorescence signals.

### CNV-seq analysis

Sample DNA was extracted using a commercial kit (Beijing Berry and Kang Biotechnology Co., Ltd.). The obtained DNA sequence data were aligned to the reference genome (hg19) to determine the uniquely mappable sequence content for each chromosome. Based on bioinformatics analysis, the coverage depth for each chromosome was calculated and converted into an index to assess the risk of chromosomal abnormalities. The identified CNVs and the genes involved were annotated using databases such as OMIM, DECIPHER, Database of Genomic Variants (DGV), and UCSC Genome Browser. Relevant literature was also reviewed *via* PubMed. According to the guidelines of the American College of Medical Genetics and Genomics (ACMG), the clinical significance of CNVs was classified into five levels: pathogenicity, potential pathogenicity, unknown clinical significance, potential benign, and benign.

## Results

### Results of G-banding chromosome karyotyping analysis

**FIGURE 2:**
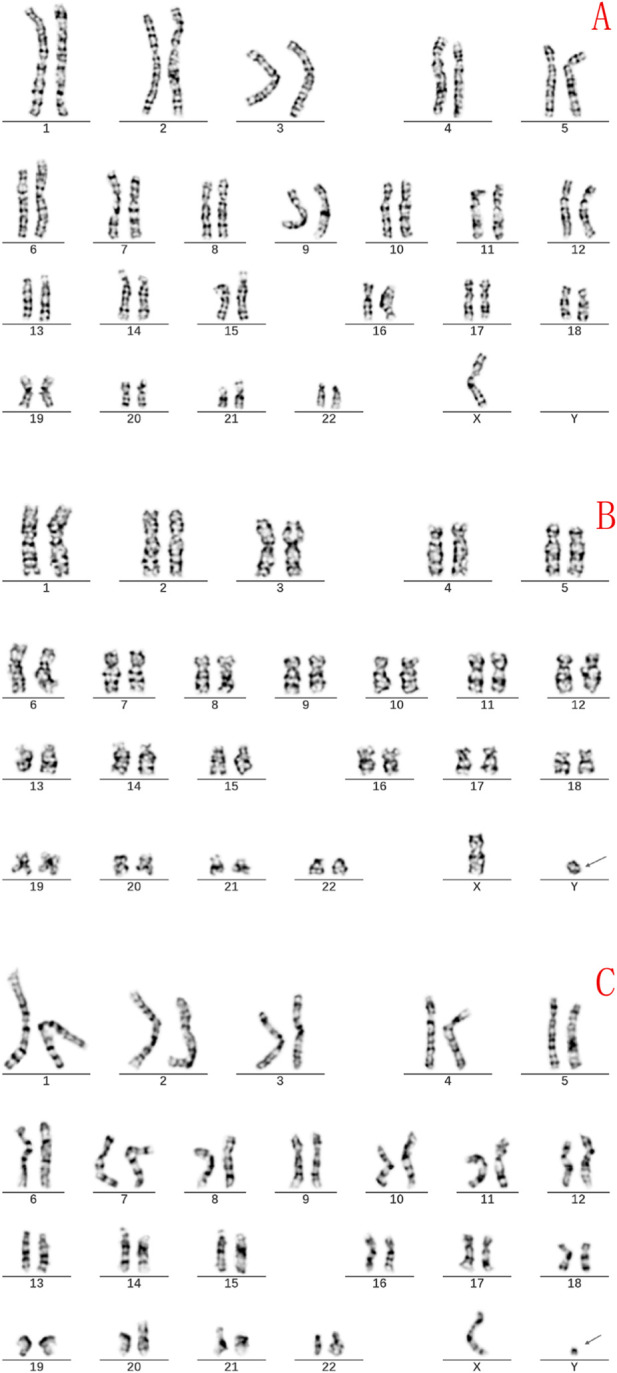
Fetal G-banding karyotype analysis reveals three types of chimeric cell lines. **(A)** A representative metaphase spread displaying a karyotype of 45, X chromosome, which is the predominant cell line (82%). **(B)** Metaphase spread showing a karyotype of 46, X, dic r(Y; Y) (13%). The dicentric Y chromosome is indicated with an arrow. **(C)** Metaphase spread showing a 46, X, r(Y) karyotype (5%). The ring-shaped Y chromosome is indicated by an arrow.

**FIGURE 3:**
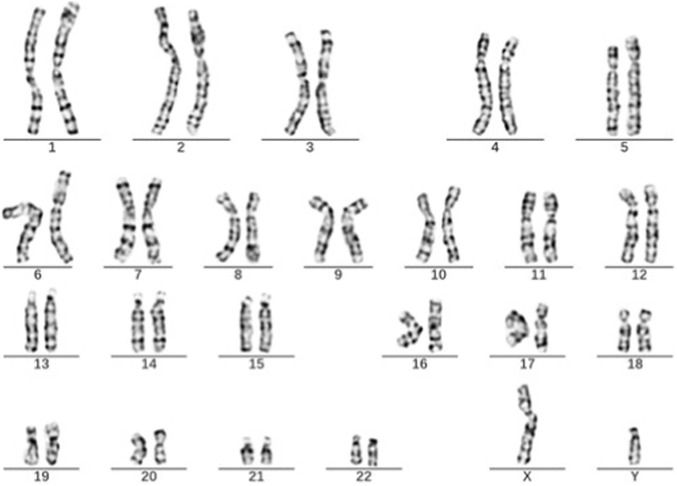
Paternal G-banding karyotype showing a normal 46, XY result. No structural or numerical abnormalities were detected, indicating that the fetal complex ring Y chromosome abnormalities arose *de novo*.

The initial G-banding karyotype of the fetus was 45,X[82]/46,X,?dic r(Y; Y)[13]/46, X,?r(Y)[5] (Figures 2A–C). This result suggested the presence of multiple cell lines, with the 45, X karyotype being predominant (82%). The mosaic karyotypes involving the ring Y chromosome, including dic r(Y; Y) and r(Y), accounted for 13% and 5%, respectively. The karyotype analysis of the fetal father’s chromosomes was 46, XY (Figure 3), indicating that the fetal karyotype arose *de novo*.

## FISH test results

**FIGURE 4:**
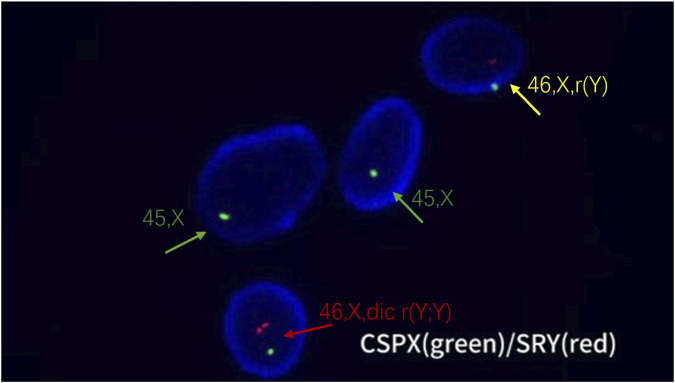
FISH analysis of cultured amniotic fluid cells using CSPX (green, X chromosome centromere) and SRY (red, Y chromosome) probes. Three types of nuclei are visible: - Nucleus with green signal only (green arrow), representing the 45, X cell line.- Nucleus with one green and one red signal (yellow arrow), representing the 46, X, r(Y) cell line.- Nucleus with one green and two red signals (red arrow), representing the 46, X, dic r(Y;Y) cell line.

The FISH results showed that the signal of the X chromosome centromere probe was normal, while the Y chromosome centromere probe exhibited signal abnormalities in some nuclei, manifested as signal loss, 1 signal, and 2 signals, which were consistent with the Y chromosome abnormalities found in G-banding karyotype analysis, further confirming the existence of a double centromere circular Y chromosome (Figure 4).

### CNV-seq detection results

**FIGURE 5:**
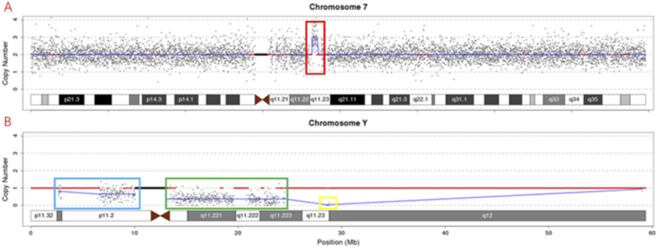
CNV-seq analysis of the fetus. **(A)** Genome-wide copy number variation plot showing a 1.40 Mb duplication at chromosome 7q11.23 (red box), corresponding to seq[hg19] dup (Li et al., 2022)(q11.23q11.23) chr7:g.72740000_74140000dup. **(B)** Enlarged view of chromosome Y showing mosaic deletions: a 7.44 Mb deletion at p11.31-p11.2 (copy number 0.7, blue box), an 11.40 Mb deletion at q11.1-q11.223 (copy number 0.4, green box), and a 0.28 Mb deletion at q11.23 (copy number 0, yellow box).

CNV-seq detection showed a 1.40 Mb repeat region at q11.23 on chromosome 7, which is seq [hg19] dup (q11.23q11.23) chr7: g.72740000_74140000up; The chimeric deletion 7.44 Mb region (copy number 0.7) at positions p11.31-p11.2 of chromosome Y is seq [hg19] del (Y) (p11.31p11.2) chrY: g.2640000_10080000del, while the chimeric deletion 11.40 Mb region (copy number 0.4) at positions q11.1-Q11.223 is seq [hg19] del (Y) (q11.1q11.23) chrY: g.13120000-24520000del, and the deletion 0.28 Mb region (copy number 0) at position q11.23 is seq [hg19] del (Y) (q11.23q11.23) chrY: g.28520000-2880000del (Figure 5).

**FIGURE 6:**
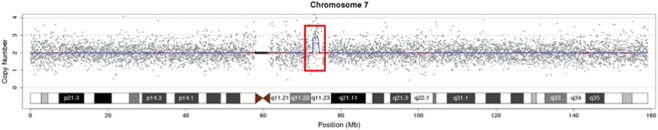
CNV-seq analysis of the father. The plot shows the same 1.40 Mb duplication at chromosome 7q11.23 (red box) as identified in the fetus (seq[hg19] dup (Li et al., 2022)(q11.23q11.23) chr7:g.72740000_74140000dup), confirming paternal inheritance of the 7q11.23 microduplication. No abnormalities were detected on the Y chromosome, consistent with the normal paternal karyotype (46,XY).

Based on the integrated results from multiple techniques, the Y chromosome breakpoints were delineated, and the final karyotype was designated as 45,X[82]/46,X,dic r(Y; Y)(p11.31q11.23; p11.31q11.23)[13]/46,X,r(Y)(p11.31q11.23)[5]dn.The maternal CNV-seq result was normal. However, the paternal CNV-seq analysis revealed a 1.40 Mb duplication at chromosome 7q11.23, designated as seq[hg19] dup (7)(q11.23q11.23) chr7:g.72740000_74140000dup, indicating that the duplication identified in the fetus at 7q11.23 was paternally inherited (Figure 6).

### Genetic counseling and pregnancy outcomes

Based on the comprehensive results of G-banding karyotyping, FISH, CNV-seq, and ultrasound findings, genetic counseling was provided to the couple. The normal paternal karyotype indicated that the fetal chromosomal abnormalities occurred *de novo*, suggesting a low recurrence risk. Informed that the pregnant woman had a chimera of fetus 45, X [82]/46, X, dic r (Y; Y) (p11.31q11.23; p11.31q11.23) [13]/46, X, r (Y) (p11.31q11.23) [5] dn, accompanied by a 1.40 Mb region duplication at q11.23 on chromosome 7. The fetal karyotype revealed a *de novo* variant, whereas the 1.40 Mb duplication at 7q11.23 was paternally inherited. The recurrence risk for this genetic condition in future pregnancies is 50%. It is strongly recommended that any subsequent pregnancy undergo definitive prenatal diagnosis or that the couple considers preimplantation genetic testing (PGT) to mitigate this risk. They were counseled that, despite the absence of obvious ultrasound abnormalities, the specific genetic changes could pose potential risks to fetal development. The eventual clinical manifestations were explained to depend on the structure, breakpoints, and degree of mosaicism of the RCs. Due to the loss of significant portions of the Y chromosome, the phenotypic spectrum was outlined, ranging from Turner syndrome stigmata in females, ambiguous external genitalia, and gonadal dysgenesis with infertility, to a completely normal male appearance. At the same time, the 1.40 Mb region is duplicated at q11.23 on chromosome 7, involving 22 protein coding genes. Patients with this syndrome may experience varying degrees of intellectual disability, language delay, congenital heart disease, varying degrees of ventricular dilation, thin corpus callosum, cerebellar hypoplasia, unilateral renal hypoplasia, spinal abnormalities, cryptorchidism, anxiety disorders, selective mutism, attention deficit hyperactivity disorder (ADHD), autism spectrum disorders, *etc.* They may also show similar manifestations to their fathers. The long-term prognosis regarding sexual development and potential treatment options after birth were also discussed. Following comprehensive genetic counseling and full disclosure of the fetal condition and associated risks, the final decision regarding pregnancy continuation was made by the couple based on informed consent. The pregnancy resulted in the spontaneous vaginal delivery of a live male infant on 27 June 2024, at 35 weeks and 5 days of gestation. At birth, the infant measured 48 cm in length and weighed 2,800 g, with no obvious phenotypic abnormalities observed. Postnatal follow-up indicated that at birth, the infant’s length and weight were in the low-to-average range. While both parameters showed catch-up growth by 1 month of age, by 3 months of age, the infant’s weight had declined, approaching −3 standard deviations (SD) below the mean. From 3 to 8 months of age, linear growth demonstrated good catch-up, reaching an average level, and weight approached the average range. However, after 8 months of age, weight gain was unsatisfactory. By 1 year of age, weight had decreased to −2 SD (below average) and length had fallen to the low-to-average range, leading to a diagnosis of malnutrition (underweight). At a recall assessment at 1 year and 7 months of age, the child’s physical examination revealed a length of 76 cm (low-to-average, −2.79 SDS) and a weight of 9.4 kg (average to low, −1.91 SDS). Clinical manifestations included mild language developmental delay; although the child could walk independently, spontaneous speech was limited to a few reduplicated words and single syllables. Physical examination findings included a rounded nasal tip, a short philtrum, a unilateral single transverse palmar crease on the left hand, and a right inguinal mass, soft in consistency, measuring approximately 5 × 2 cm. Both testes were non-palpable in the scrotum. No intellectual disability was evident. Laboratory investigations, including sex hormone profiling, yielded results within normal ranges (Prog 0.1 ng/mL, E2 10 pg/mL, PRL 4.17 ng/mL, Testo 0.13 ng/mL, hFSH 0.87 mIU/mL, hLH 0.04 mIU/mL). Y-chromosome microdeletion analysis showed no deletions at the tested AZF loci: AZFa (sY86, sY84), AZFb (sY134, sY127), and AZFc (sY254, sY255). A color Doppler ultrasound of the scrotum and inguinal regions revealed a right funicular hydrocele and bilateral cryptorchidism, with the right testis measuring 12 × 10 × 6 mm and the left testis measuring 11 × 10 × 7 mm. Given the patient’s young age, long-term follow-up through puberty is warranted to assess gonadal development and future reproductive function.

## Discussion

RCs is aberrant chromosomes that can originate from one or more chromosomes. It refers to a break in the distal ends of the long and short arms of a chromosome, where the broken ends of the long and short arms containing a centromere segment meet to form a circular chromosome, and the segment without a centromere is lost. Its clinical manifestations depend on the size, origin, location of break points, and degree of cellular mosaic of the circular chromosome (Ravel and Siffroi, 2009). Among these, ring Y chromosome (RCY) is exceptionally rare, accounting for only 6% of all ring chromosome cases (Li et al., 2022). Patients with RCY may exhibit a wide spectrum of clinical phenotypes. Of the 59 RCY cases with known sex assignment, 48 presented as phenotypic males and 11 as phenotypic females (DuPont et al., 2024). In phenotypic males, the most common presentation is a normal male phenotype with infertility. Serena Chong et al. reported a case of a 27-year-old male with mosaic ring Y chromosome (46, X, r(Y)/45, X) who presented with severe oligozoospermia but had a largely normal phenotype (Chong and Muir, 2025). Other clinical presentations include short stature with infertility, Turner-like features such as cubitus valgus, widely spaced nipples, webbed neck, and skeletal anomalies, and less commonly, ambiguous genitalia (Dong et al., 2014). All reported phenotypic females presented with primary amenorrhea and Turner syndrome-like features, including short stature, shield chest, lymphedema, and streak gonads (DuPont et al., 2024). In adults, testosterone levels may be normal or low, typically accompanied by normal or elevated gonadotropin levels, normal or reduced testicular size, and severe oligozoospermia or azoospermia (Dong et al., 2014).

The clinical manifestations of RCY patients are wide-ranging, influenced by the presence or absence of key Y chromosome genes/loci and the degree of chimerism in the 45X cell line. Due to the mitotic instability of RCY, chimerism in the 45, X cell line is common. The clinical impact of RCY is not only influenced by the proportions of 46, X, r(Y), and 45, X cell lines, but also by their distribution in different tissues (Chong and Muir, 2025).

The initial diagnosis result of the G-banding karyotype of the fetus in this study was 45, X[82]/46,X,? dic r(Y; Y)[13]/46,X,? r(Y)[5]dn,The chromosomal karyotype analysis of the father of the fetus showed no abnormalities, indicating that the fetus is a newly developed variant with a low risk of genetic recurrence. This karyotype suggests the presence of multiple cell lines in the fetus, presenting a complex chimeric state, with the 45, X karyotype being the predominant (82%), while there is also a chimeric karyotype composed of double centromere circular Y chromosomes, including dic r (Y; Y) and r (Y), accounting for 13% and 5%, respectively. The 45, X karyotype is a hallmark of Turner syndrome, and patients typically exhibit various clinical features such as short stature, gonadal dysgenesis, cardiovascular malformations, and renal anomalies. In our case, this phenomenon can be explained by two mechanisms. First, tissue-specific mosaicism, where a significant proportion of 45, X cells may contribute to impaired testicular function. Second, the mitotic instability of the ring Y chromosome, through mechanisms such as sister chromatid exchange, can lead to the formation of dicentric rings (dic r(Y;Y)) and subsequent anaphase lag and chromosome loss. This well-established model explains the origin of the predominant 45, X cell line and the complex mosaicism observed in our patient. Zheng et al., (2019) believe that although most dic r(Y; Y) patients have normal fetal phenotypes, abnormal phenotypes caused by sex chromosome aberrations and chimerism gradually appear after birth, especially during puberty. The finding of bilateral cryptorchidism at 19 months confirms a degree of testicular dysfunction or impaired descent. This is a critical finding, as the presence of a 45, X cell line in individuals with ring Y chromosome mosaicism has been associated with gonadal anomalies and an increased risk of gonadoblastoma. Case reports have documented gonadoblastoma in both a boy with bilateral cryptorchidism and a girl with streak gonads who carried a mosaic ring Y chromosome karyotype (Layman et al., 2009; Khudr and Benirschke, 1973). This evidence strongly supports our recommendation for long-term, ongoing surveillance, including regular physical examinations, scrotal/abdominal imaging (ultrasound), and monitoring of serum tumor markers, particularly as the patient progresses through puberty.

FISH technology uses specific probe hybridization to visually display signal abnormalities of Y chromosome centromere probes in some cell nuclei, manifested as signal loss, 1 signal, and 2 signals, which is consistent with the Y chromosome abnormalities found in G-banding karyotype analysis. This further confirms the existence of double centromere circular Y chromosomes and provides strong supplementary evidence for chromosome karyotype analysis, especially in detecting chromosomal microstructural variations and numerical abnormalities with high sensitivity and specificity (Chrzanowska et al., 2020). CNV-seq screened for chromosome copy number variations at the whole genome level, showing a chimeric deletion of 7.44 Mb region (copy number 0.7) at p11.31-p11.2, a chimeric deletion of 11.40 Mb region (copy number 0.4) at q11.1-q11.223, and a deletion of 0.28 Mb region (copy number 0) at q11.23 on the Y chromosome. However, the inability to detect Y chromosome repeat fragments may be related to the limitations of this technology, which makes it difficult to detect chimeras with varying proportions of normal and abnormal cell lines (Peng and Yuan, 2018; Ma et al., 2021). Simultaneously detected a 1.40 Mb repeat region at q11.23 on chromosome 7,which was definitively inherited from the father. CNV-seq technology makes the detection of fetal genomic variations more comprehensive and accurate, which helps to gain a deeper understanding of the genetic mechanisms and potential pathogenic factors of chromosomal abnormalities. Finally, by combining multiple techniques for detection, the Y chromosome breakpoint was identified, and the final chromosome karyotype result was determined to be 45, X[82]/46,X,dic r(Y; Y)(p11.31q11.23; p11.31q11.23)[13]/46,X,r(Y)(p11.31q11.23)[5]dn. Although G-banding chromosome karyotyping analysis remains the “gold standard” for chromosome disease detection, it failed to detect the 1.40 Mb duplication at q11.23 on chromosome 7, the 11.40 Mb fragment of chimeric deletion at q11.1-q11.223 on chromosome Y, and the p11.31q11.23 breakpoint in this case, which is related to its technical limitations (Huang et al., 2022). Therefore, it is necessary to combine G-banding chromosome karyotyping analysis, FISH, and CNV-seq techniques for detection.

In this study, CNV-seq showed a 1.40 Mb repeat region at q11.23 on chromosome 7, involving 22 protein coding genes. After querying the ClinGen database resources, this fragment covers approximately 99.8% of the recurrent (Williams Beuren syndrome) region (including ELN) on chromosome 7, and involves all protein coding genes in this region. There is sufficient evidence (Triplosensitivity Score: 3) to indicate that patients with three times the dose in this region exhibit the clinical phenotype of WILLIAMS-BEUREN REGION DUPLIcation SYNDROME (Lechich et al., 2020; Klein-Tasman and Mervis, 2018; Wang et al., 2023; Mervis et al., 2015; Klein-Tasman et al., 2022). The main clinical symptoms of this syndrome include varying degrees of intellectual disability and delayed language development. Craniofacial malformation, congenital heart disease (heart defect, patent ductus arteriosus, aortic dilation), diaphragmatic hernia, joint laxity, decreased muscle tone, brain (MRI) Abnormalities, varying degrees of ventricular dilation, thin corpus callosum, underdeveloped cerebellar gyrus, unilateral renal agenesis, spinal abnormalities, cryptorchidism, anxiety disorders (especially social anxiety), selective mutism, attention deficit hyperactivity disorder (ADHD), autism spectrum disorders(ASD), *etc.* Approximately 30% of patients with 7q11.23 microduplication syndrome have one or more congenital malformations.

The 7q11.23 duplication region is an important genomic region whose copy number variants (CNVs) are closely associated with various neurodevelopmental disorders. *GTF2I* is a key gene within the 7q11.23 duplication region (López-Tobón et al., 2023; Pinelli et al., 2020). Pinelli et al. described a family carrying a small 7q11.23 duplication involving *GTF2I* that segregated with mild cognitive impairment across three generations. They demonstrated that *GTF2I* expression was elevated in peripheral blood mononuclear cells of affected individuals, consistent with findings in subjects with typical Dup7 (Pinelli et al., 2020). This provides strong evidence that *GTF2I* is a critical gene responsible for the cognitive impairment and neurodevelopmental features of 7q11.23 duplication syndrome. In our patient, the 1.40 Mb duplication includes *GTF2I*, and the mild language delay observed at 19 months may represent an early manifestation of the neurodevelopmental phenotype associated with *GTF2I* overexpression.

Recent studies suggest that the gene dosage of *GTF2I* regulates neuronal differentiation and social behavior (López-Tobón et al., 2023). In 7q11.23 microduplication syndrome, duplication of *GTF2I* leads to abnormal neuronal differentiation and connectivity, which is closely related to the cognitive and behavioral impairments observed in affected individuals (López-Tobón et al., 2023). Mouse model studies have also supported the role of *GTF2I* in social behavior; for example, increased dosage of *Gtf2i* has been shown to result in excessive sociability and reduced fear response (López-Tobón et al., 2023). The dosage effect of *GTF2I* not only influences neurodevelopment but may also be associated with autism spectrum disorder (ASD) (Cupaioli et al., 2021; Qaiser et al., 2021). The 7q11.23 duplication is one of the most common recurrent copy number variants associated with ASD, yet not all individuals carrying this duplication exhibit an ASD phenotype, suggesting the involvement of additional genetic factors or modifier genes (Qaiser et al., 2021; Goh et al., 2025).

The *ELN* gene encodes elastin, a critical component of vascular and connective tissue. In Williams-Beuren syndrome (WBS), haploinsufficiency of *ELN* is the primary cause of cardiovascular abnormalities, such as supravalvular aortic stenosis (Alesi et al., 2021; Evangelidou et al., 2020). Although *ELN* is duplicated rather than deleted in 7q11.23 duplication syndrome, its overexpression may also have an impact on the cardiovascular system (Wei et al., 2024; Tcheandjieu et al., 2022). For instance, one study noted that Williams syndrome is associated with precocious puberty, whereas 7q11.23 duplication syndrome is associated with delayed puberty, potentially reflecting the effect of gene dosage in this region on hormonal regulation. Our patient’s physical features, including the short philtrum and rounded nasal tip, may be related to altered ELN expression, although long-term cardiovascular follow-up is warranted.

The chimeric deletion of 7.44 Mb region (copy number 0.7) at positions p11.31-p11.2, 11.40 Mb region (copy number 0.4) at positions q11.1-q11.223, and 0.28 Mb region (copy number 0) at position q11.23 on the Y chromosome cover the entire region of the Y chromosome, possibly indicating the phenotype of 45, XO/46, XY Mosaic Intersex Syndrome. According to public databases, 45, XO/46, XY Mosaic Intersex Syndrome may have some clinical symptoms of Turner syndrome (such as short stature, gonadal dysplasia, *etc.*), and the patient phenotype is related to the ratio of chimerism between the two types of cells, ranging from close to normal male to fully displaying Turner syndrome phenotype. In addition, some patients may exhibit abnormalities in their external genitalia, as well as pseudohermaphroditism (Gerli et al., 1976; Jackson et al., 1966).

The *SRY* gene is located in the Yp11.3 region and is the key gene for male sex determination. Its presence initiates testicular development. Although the p11.31-p11.2 deletion encompasses the *SRY* gene, the copy number of 0.7 indicates a mosaic deletion, meaning that some cells still retain the *SRY* gene. Complete deletion of the *SRY* gene would result in a female phenotype in individuals with a 46,XY karyotype, a condition known as Swyer syndrome (Kane et al., 2023). However, even mosaic deletion can affect *SRY* gene function, leading to disorders of sex development (Faria et al., 2023). Studies have reported that deletions in the Yp11.2 region can result in the presence of Y chromosome-associated alleles in females with Turner syndrome during sex determination testing (Lai et al., 2023).

The proximal region of the *SRY* gene (Yp11.32) contains the *SHOX* gene, which has copies in the pseudoautosomal region 1 (PAR1) of both the X and Y chromosomes (Cai et al., 2023). Haploinsufficiency of the *SHOX* gene is one of the main causes of short stature in patients with Turner syndrome (Wang et al., 2022). Although the deletion in this case primarily involves the Yp region, because *SHOX* is located in the PAR1 region and is present on both the X and Y chromosomes, it undergoes recombination during male meiosis and behaves genetically like an autosomal gene. Therefore, deletion of the Yp region may affect *SHOX* gene dosage and consequently influence height.

Deletions in the Yp11.2 region may also extend into the AZFa region, which contains critical genes such as *DDX3Y* and *USP9Y*. Complete deletion of the AZFa region leads to Sertoli cell-only syndrome (SCOS) or azoospermia. *DDX3Y* is considered a key spermatogenesis factor in the AZFa region that is crucial for non-obstructive azoospermia (NOA) in humans (Dicke et al., 2023; Li et al., 2024).

The mosaic 11.40 Mb deletion at q11.1-q11.223 encompasses a large portion of the Y chromosome long arm (Yq) and very likely includes the complete AZFb and AZFc regions (McElreavey et al., 2007). Complete deletion of AZFb typically results in irreversible spermatogenic arrest, usually presenting as Sertoli cell-only syndrome with azoospermia and a zero percent success rate for surgical sperm retrieval. The AZFc region is the most common type of Y chromosome microdeletion (Chernykh et al., 2022). It contains the *DAZ* (Deleted in Azoospermia) gene family (*DAZ1-DAZ4*), as well as *CDY1*, *BPY2*, *GOLGA2LY*, and other genes (Nakagawa et al., 2023; Ou et al., 2024). The *DAZ* genes are primate-specific and are closely involved in the translational regulation of mRNAs related to cell proliferation during spermatogenesis. Deletion of *DAZ* genes may lead to defective proliferation of c-KIT positive spermatogonia and spermatogenic failure (Ou et al., 2024). Although AZFc deletion severely impairs spermatogenesis, it does not always completely abolish it, and some patients may still have sperm successfully retrieved through microdissection testicular sperm extraction (micro-TESE).

The copy number of 0.4 indicates a mosaic deletion in this region, meaning that the genes in this region are deleted in some cells but present in others. This mosaic ratio can significantly influence the clinical phenotype, with higher levels of mosaicism correlating with more severe reproductive dysfunction.

The complete deletion (copy number 0) of the q11.23 region indicates that all cells have lost this portion of genetic material. This region is located distal to AZFc and may contain the boundary genes of AZFc as well as portions of the *DAZ/CDY* gene clusters (Nakagawa et al., 2023; Ou et al., 2024).

In summary, these mosaic or complete deletions in the Y chromosome p11.31-p11.2, q11.1-q11.223, and q11.23 regions may lead to altered dosage or loss of multiple critical genes, including *SRY*, *SHOX*, *DDX3Y*, and *USP9Y*, resulting in severe male infertility and potentially other developmental abnormalities. In-depth analysis of the molecular characteristics of these deletions has important guiding significance for clinical diagnosis, genetic counseling, and the development of personalized treatment strategies, particularly in the context of assisted reproduction.

## Conclusion

This study conducted genetic analysis on a rare and complex chromosome karyotype 45, X [82]/46, X, dic r (Y; Y) (p11.31q11.23; p11.31q11.23) [13]/46, X, r (Y) (p11.31q11.23) [5] dn chimeric fetus, and comprehensively used G-banding chromosome karyotyping analysis, FISH, and CNV seq techniques to clarify the chromosomal abnormalities and genetic characteristics of the fetus, and preliminarily explored their association with clinical phenotypes. This multi technology joint detection method provides comprehensive and accurate information for the diagnosis and research of rare chromosomal diseases, which helps to provide stronger basis for clinical genetic counseling and prenatal diagnosis, and also lays the foundation for further in-depth research on the pathogenesis and genetic laws of such diseases. For similar rare cases of chromosomal abnormalities, multidisciplinary collaboration should be strengthened to comprehensively consider genetic factors, clinical phenotypes, and prognosis, and provide personalized genetic counseling and medical advice for families to improve the quality of birth and reduce the incidence of birth defects.

## Data Availability

The original contributions presented in the study are included in the article/supplementary material, further inquiries can be directed to the corresponding authors.

## References

[B1] Alesi V. Loddo S. Orlando V. Genovese S. Di Tommaso S. Liambo M. T. (2021). Atypical 7q11.23 deletions excluding ELN gene result in Williams-Beuren syndrome craniofacial features and neurocognitive profile. Am. J. Med. Genet. A 185 (1), 242–249. 10.1002/ajmg.a.61937 33098373

[B2] Blanco J. Farreras A. Egozcue J. Vidal F. (2003). Meiotic behavior of the sex chromosomes in a 45, X/46, X, r(Y) pstient whose semen was assessed by fluorescent *in situ* hybridization. Fertil. Steril. 79 (4), 913–918. 10.1016/s0015-0282(02)04931-2 12749430

[B3] Cai M. Chen X. Li Y. Lin N. Huang H. Xu L. (2023). Genetic analysis, ultrasound phenotype, and pregnancy outcomes of fetuses with Xp22.33 or Yp11.32 microdeletions. J. Perinat. Med. 52 (1), 96–101. 10.1515/jpm-2023-0190 37846158

[B4] Chernykh V. B. Ryzhkova O. P. Kuznetsova I. A. Kazaryan M. S. Sorokina T. M. Kurilo L. F. (2022). Deletions in AZFc region of Y chromosome in Russian fertile men. Russ. J. Genet. 58 (7), 850–856. 10.1134/s1022795422070043

[B5] Chong S. Muir C. A. (2025). Ring Y chromosome as an unusual cause of severe oligozoospermia. Endocrinol. Diabetes Metab. Case Rep. 2025 (4), e250113. 10.1530/EDM-25-0113 41277627 PMC12659933

[B6] Chrzanowska N. M. Kowalewski J. Lewandowska M. A. (2020). Use of Fluorescence *in situ* Hybridization (FISH) in diagnosis and tailored therapies in solid tumors. Molecules 25 (8), 1864. 10.3390/molecules25081864 32316657 PMC7221545

[B7] Colindres J. V. Axelrad M. McCullough L. Smith E. O. B. Huang G. O. Tu D. D. (2016). Evidence-based management of patients with 45, X/46, XY gonadal dysgenesis and male sex assignment: from infancy to adulthood. Pediatr. Endocrinol. Rev. 13 (3), 585–601. 27116846

[B8] Cupaioli F. A. Fallerini C. Mencarelli M. A. Perticaroli V. Filippini V. Mari F. (2021). Autism spectrum disorders: analysis of Mobile elements at 7q11.23 Williams-Beuren Region by comparative genomics. Genes (Basel). 12 (10), 1605. 10.3390/genes12101605 34680999 PMC8535890

[B9] Dicke A. K. Pilatz A. Wyrwoll M. J. Punab M. Ruckert C. Nagirnaja L. (2023). DDX3Y is likely the key spermatogenic factor in the AZFa region that contributes to human non-obstructive azoospermia. Commun. Biol. 6 (1), 350. 10.1038/s42003-023-04714-4 36997603 PMC10063662

[B10] Dong Y. Yu X. Wang R. 45 Li L. L. Jiang Y. T. Liu R. Z. (2014). X/46, X,r(Y)/46, x,dic r(Y) karyotype in an azoospermic male: a case report. Cytogenet Genome Res. 142 (2), 140–144. 10.1159/000356467 24335108

[B11] DuPont B. R. Li P. Liehr T. (2024). “Human ring chromosomes: a practical guide for Clinicians and families,” in Human ring chromosomes: a practical guide for Clinicians and families. Editors Li P. Liehr T. (Cham: Springer International Publishing), 347–352.

[B12] Evangelidou P. Kousoulidou L. Salameh N. Alexandrou A. Papaevripidou I. Alexandrou I. M. (2020). An unusual combination of an atypical maternally inherited novel 0.3 Mb deletion in Williams-Beuren region and a *de novo* 22q11.21 microduplication in an infant with supravalvular aortic stenosis. Eur. J. Med. Genet. 63 (12), 104084. 10.1016/j.ejmg.2020.104084 33045407

[B13] Faria J. A. D. Jr Moraes D. R. Kulikowski L. D. Batista R. L. Gomes N. L. Nishi M. Y. (2023). Cytogenomic investigation of syndromic Brazilian patients with differences of sexual development. Diagn. (Basel) 13 (13), 2235. 10.3390/diagnostics13132235 37443631 PMC10340279

[B14] Gerli M. Biagioni M. Migliorini Bruschelli G. Ferrarese R. Rosi G. (1976). A case of true hermaphroditism with 45X/46XY mosaicism. Hum. Genet. 34 (1), 93–97. 10.1007/BF00284444 987015

[B15] Goh S. Thiyagarajan L. Dudding-Byth T. Pinese M. Kirk E. P. (2025). A systematic review and pooled analysis of penetrance estimates of copy-number variants associated with neurodevelopment. Genet. Med. 27 (1), 101227. 10.1016/j.gim.2024.101227 39092588

[B16] Houk C. P. Lee P. A. (2008). Consensus statement on terminology and management: disorders of sex development. Sex. Dev. 2 (4-5), 172–180. 10.1159/000152032 18987491

[B17] Huang Y. Huang Q. Liu J. Guo M. Liu Y. Lai D. (2022). Concurrent ovarian and tubal ectopic pregnancy after IVF-ET: case report and literature review. Front. Physiol. 13, 850180. 10.3389/fphys.2022.850180 35444560 PMC9013932

[B18] Jackson W. P. Hoffman M. Makda H. (1966). The 45XO/46XY mosaic intersex syndrome. J. Med. Genet. 3 (1), 23–32. 10.1136/jmg.3.1.23 5911827 PMC1012889

[B19] Kane D. T. Dempsey M. A. Burke A. L. Morrison J. J. (2023). Prenatal diagnosis and fetal sonographic features of Swyer syndrome. J. Fetal Med. 10 (1), 36–39. 10.1055/s-0043-57035

[B20] Khudr G. Benirschke K. (1973). Y ring chromosome associated with gonadoblastoma *in situ* . Obstet. Gynecol. 41 (6), 897–901. 4708485

[B21] Klein-Tasman B. P. Mervis C. B. (2018). Autism spectrum symptomatology among children with duplication 7q11.23 syndrome. J. Autism Dev. Disord. 48 (6), 1982–1994. 10.1007/s10803-017-3439-z 29307037 PMC6003247

[B22] Klein-Tasman B. P. Yund B. D. Mervis C. B. (2022). The behavioral phenotype of 7q11.23 duplication syndrome includes risk for oppositional behavior and aggression. J. Dev. Behav. Pediatr. 43 (6), e390–e398. 10.1097/DBP.0000000000001068 35580312 PMC9329151

[B23] Lai L. Huang X. L. Mei D. R. Li Y. Wu Y. C. (2023). Analysis of a Yp11.2 region deletion in a Chinese female with Turner syndrome: a case report. Heliyon 9 (4), e15162. 10.1016/j.heliyon.2023.e15162 37089332 PMC10113852

[B24] Layman L. C. Tho S. P. Clark A. D. Kulharya A. McDonough P. G. (2009). Phenotypic spectrum of 45, X/46, XY males with a ring Y chromosome and bilaterally descended testes. Fertil. Steril. 91 (3), 791–797. 10.1016/j.fertnstert.2007.12.078 18555994 PMC3888814

[B25] Lechich K. M. Zarate Y. A. Daily J. A. Collins R. T. (2020). Aortic geometry in patients with duplication 7q11.23 compared to healthy CPhenotypic spectrum of 45,X/46,XY males with a ring Y chromosome and bilaterally descended testesontrols. Pediatr. Cardiol. 41 (6), 1199–1205. 10.1007/s00246-020-02375-2 32474735

[B26] Li P. Dupont B. Hu Q. Crimi M. Shen Y. Lebedev I. (2022). The past, present, and future for constitutional ring chromosomes: a report of the international consortium for human ring chromosomes. Hum. Genet. Genom Adv. 3 (4), 100139. 10.1016/j.xhgg.2022.100139 36187226 PMC9519620

[B27] Li J. Song S. Zhang J. (2024). Where are the formerly Y-linked genes in the Ryukyu spiny rat that has lost its Y chromosome? Genome Biol. Evol. 16 (3), evae046. 10.1093/gbe/evae046 38478711 PMC10959550

[B28] López-Tobón A. Shyti R. Villa C. E. Cheroni C. Fuentes-Bravo P. Trattaro S. (2023). GTF2I dosage regulates neuronal differentiation and social behavior in 7q11.23 neurodevelopmental disorders. Sci. Adv. 9 (48), eadh2726. 10.1126/sciadv.adh2726 38019906 PMC10686562

[B29] Ma N. Xi H. Chen J. Peng Y. Jia Z. Yang S. (2021). Integrated CNV-seq, karyotyping and SNP-array analyses for effective prenatal diagnosis of chromosomal mosaicism. BMC Med. Genomics 14 (1), 56. 10.1186/s12920-021-00899-x 33632221 PMC7905897

[B30] McElreavey K. Ravel C. Houate B. E. Mandelbaum J. Chantot-Bastaraud S. Siffroi J. P. (2007). “Y chromosome microdeletions and haplotypes,” in The genetics of Male infertility (Totowa, NJ: Humana Press), 239–249. 10.1007/978-1-59745-176-5_15

[B31] Melzer P. S. Guan X. Y. Trent J. M. (1993). Telomere capture stabilizes chromosome breakage. Nat. Genet. 4 (3), 252–255. 10.1038/ng0793-252 8358433

[B32] Mervis C. B. Klein-Tasman B. P. Huffman M. J. Velleman S. L. Pitts C. H. Henderson D. R. (2015). Children with 7q11.23 duplication syndrome: psychological characteristics. Am. J. Med. Genet. A 167 (7), 1436–1450. 10.1002/ajmg.a.37071 25900101 PMC4545595

[B33] Nakagawa Y. Tada A. Kojo K. Tsuchiya H. Kurobe M. Uchida M. (2023). Analysis of the correlation between gene copy deletion in the AZFc region and male infertility in Japanese men. Reprod. Biol. 23 (1), 100728. 10.1016/j.repbio.2022.100728 36640629

[B34] Ou N. Wang Y. Xu S. Luo J. Zhang C. Zhang Y. (2024). Primate-Specific DAZ regulates translation of cell proliferation-related mRNAs and is essential for maintenance of Spermatogonia. Adv. Sci. (Weinh) 11 (29), e2400692. 10.1002/advs.202400692 38783578 PMC11304246

[B35] Peng J. P. Yuan H. M. (2018). Application of chromosomal microarray analysis for a cohort of 2600 Chinese patients with miscarriage. Hereditas 40 (9), 779–788. 10.16288/j.yczz.18-120 30369481

[B36] Pinelli M. Terrone G. Troglio F. Squeo G. M. Cappuccio G. Imperati F. (2020). A small 7q11.23 microduplication involving GTF2I in a family with intellectual disability. Clin. Genet. 97 (6), 940–942. 10.1111/cge.13753 32349160 PMC7318190

[B37] Pristyazhnyuk I. E. Menzorov A. G. (2018). Ring chromosomes: from formation to clinical potential. Protoplasma 255 (2), 439–449. 10.1007/s00709-017-1165-1 28894962

[B38] Qaiser F. Yin Y. Mervis C. B. Morris C. A. Klein-Tasman B. P. Tam E. (2021). Rare and low frequency genomic variants impacting neuronal functions modify the Dup7q11.23 phenotype. Orphanet J. Rare Dis. 16 (1), 6. 10.1186/s13023-020-01648-6 33407644 PMC7788915

[B39] Ravel C. Siffroi J. P. (2009). Y chromosome structural abnormalities and Turner’s syndrome. Gynecol. Obstet. Fertil. 37 (6), 511–518. 10.1016/j.gyobfe.2009.04.018 19464936

[B40] Tcheandjieu C. Xiao K. Tejeda H. Lynch J. A. Ruotsalainen S. Bellomo T. (2022). High heritability of ascending aortic diameter and trans-ancestry prediction of thoracic aortic disease. Nat. Genet. 54 (6), 772–782. 10.1038/s41588-022-01070-7 35637384 PMC13102105

[B41] Wang H. Liang B. Wang Y. Huang H. Lin N. Xu L. (2022). Retrospective analysis of the sex chromosomal copy number variations in 186 fetuses using single nucleotide polymorphism arrays. Front. Genet. 13, 997757. 10.3389/fgene.2022.997757 36531253 PMC9751192

[B42] Wang Y. Liu C. Hu R. Geng J. Lu J. Zhao X. (2023). Intrauterine phenotype features of fetuses with 7q11.23 microduplication syndrome. Orphanet J. Rare Dis. 18 (1), 305. 10.1186/s13023-023-02923-y 37759207 PMC10523695

[B43] Wei S. M. Gregory M. D. Nash T. de Abreu E Gouvêa A. Mervis C. B. Cole K. M. (2024). Altered pubertal timing in 7q11.23 copy number variations and associated genetic mechanisms. iScience 27 (3), 109113. 10.1016/j.isci.2024.109113 38375233 PMC10875153

[B44] Zheng J. Yang X. Lu H. Guan Y. Yang F. Xu M. (2019). Prenatal diagnosis of sex chromosome mosaicism with two marker chromosomes in three cell lines and a review of the literature. Mol. Med. Rep. 19 (3), 1791–1796. 10.3892/mmr.2018.9798 30592288

